# Expanding Horizons in Parkinson’s Disease: Towards Integrated Care and Comprehensive Research

**DOI:** 10.3390/jcm14020385

**Published:** 2025-01-09

**Authors:** Alejandro Rodríguez-Molinero

**Affiliations:** Research Department, Consorci Sanitari de l’Alt-Penedès i Garraf, 08720 Barcelona, Spain; arodriguez@csapg.cat

Parkinson’s disease (PD) is the second most common neurodegenerative disorder worldwide and the fastest-growing neurological condition in terms of prevalence, disability, and mortality [1,2]. The economic burden associated with this disease is projected to exceed USD 79 billion in the United States by 2037, underlining the need for continued research and innovation in its management [3].

In the past decade, significant advances have been made in our understanding of Parkinson’s disease, including improvements in genetic knowledge. These developments have shed light on critical mechanisms such as mitochondrial regulation, lysosomal function, and immune system involvement, providing new insights into potential therapeutic strategies. This progress has been instrumental in fostering personalized medicine and exploring targeted treatments [4].

Efforts to improve motor symptom management remain a cornerstone of PD research. Enhanced formulations of L-dopa and advanced pharmacological therapies, such as continuous infusion treatments, aim to reduce motor fluctuations and dyskinesias. Meanwhile, emerging therapeutic targets, including glutamate modulators [5] and adenosine A2A antagonists [6], offer new avenues for disease control. Innovations in neurosurgical interventions, such as adaptive deep brain stimulation (DBS) and focused ultrasound for refractory tremors, have also broadened treatment options. Additionally, experimental approaches like gene therapy and stem cell treatments continue to show promise.

Despite these innovations, no treatment has yet been found to halt or reverse the progression of Parkinson’s disease. This stark reality underscores the importance of comprehensive patient care strategies that integrate pharmacological and non-pharmacological approaches. Lifestyle interventions, including regular physical activity, a balanced diet, and adequate sleep, play a crucial role in improving outcomes. Exercise, in particular, has demonstrated robust benefits in slowing motor decline, enhancing non-motor symptoms, and improving overall quality of life [7,8]. Among the novel rehabilitative interventions, techniques aimed at improving gait and balance are gaining attention. In this regard, this Special Issue features the work of Pollet et al., who tested the efficacy of custom-made insoles on function, balance, and quality of life through a randomized, triple-blind, controlled clinical trial. This study underscores the need for the robust validation of new non-pharmacological approaches, particularly those that address complex motor impairments.

The focus of PD research, however, is not limited to motor symptoms. In recent years, research on biomarkers for Parkinson’s disease has also gained significant attention, as they can help to improve the definition and classification of the disease. Advances in imaging techniques and biochemical biomarkers, particularly those related to oxidative stress, mitochondrial dysfunction, neuroinflammation, and Lewy body pathology, are opening up new possibilities for a more precise classification of Parkinson’s disease [9]. These advancements may also facilitate earlier detection, which is crucial for implementing interventions during the prodromal phase when neuronal damage might still be minimized.

Interestingly, non-motor symptoms can also be considered early biomarkers of Parkinson’s disease. While motor symptoms typically emerge after substantial neuronal loss has occurred, certain non-motor symptoms, such as olfactory dysfunction, rapid eye movement sleep behavior disorder (RBD), or constipation, may precede the motor syndrome by years, making them valuable clinical biomarkers during the prodromal phase. Research aimed at deepening our understanding of non-motor symptoms, their classification, and their differential expression in various patient subgroups is essential for advancing early diagnosis, disease comprehension, prognosis, and treatment strategies. While non-motor symptoms can impact quality of life as much as motor symptoms, research has historically focused more on the latter, particularly outside the most studied populations in the developed world.

This Special Issue makes significant contributions to important gaps in non-motor symptoms knowledge. Heimrich et al. contribute a comprehensive analysis of depressive symptoms, identifying specific aspects that most significantly impact patients’ quality of life, while Chmiel et al. explore the potential of transcranial direct current stimulation (tDCS) as a treatment for depression in Parkinson’s disease. Similarly, Maher et al. provide an overview of available therapies for apathy, another prevalent non-motor symptom that significantly diminishes patients’ motivation and engagement in daily life.

Beyond mood disorders, Petrijan et al. provide a valuable contribution to this Special Issue by refining the classification of non-motor symptoms. In addition, Huang et al. address autonomic dysfunction, presenting an innovative, cost-effective alternative to the sudomotor axon reflex test for evaluating autonomic dysfunction. This development is particularly relevant for low-income countries, where access to advanced diagnostic tools remains limited. By addressing diagnostic inequities, this work has the potential to improve outcomes for underserved populations globally.

Finally, Santos-García et al. explore sex differences in the expression of motor and non-motor symptoms. Their findings deepen our understanding of how gender influences the clinical presentation of Parkinson’s disease, laying the foundation for more personalized and equitable treatment strategies. Differences in symptomatology between male and female patients, if systematically understood, could lead to more tailored interventions that account for these variations.

By addressing both motor and non-motor aspects of Parkinson’s disease (PD), this Special Issue underscores the critical need for an integrated and comprehensive approach to understanding and managing this multifaceted condition. PD is a complex disorder that affects patients in diverse ways, with motor and non-motor symptoms often interacting to significantly impact quality of life. Tackling these challenges requires a holistic strategy that combines advances in diagnostics, innovative treatments, and supportive care tailored to individual needs. Continued research in these areas is vital to improving symptom management, enabling earlier interventions, and ultimately enhancing outcomes for patients worldwide.
